# Acute suppurative thyroiditis in a child with congenital third/fourth branchial arch anomaly: a case report

**DOI:** 10.11604/pamj.2024.48.120.38164

**Published:** 2024-07-19

**Authors:** Theodora Dermitzaki, Dimitra Liva, Ioanna Tritou, Ioannis Markakis, Maria Kokkinaki

**Affiliations:** 1 Department of Imaging, Venizelion General Hospital, Heraklion, Crete, Greece,; 2 Knossos Diagnosis, Heraklion, Crete, Greece

**Keywords:** Branchial arch, suppurative thyroiditis, neck mass, sinus track, case report

## Abstract

Third and fourth branchial arch anomalies belong to congenital lesions which are extremely rare accounting for <1-4% of branchial arch anomalies. In our case, a 4-year-old boy was admitted to our hospital with a painful mass on the left side of his neck. The imaging and clinical findings are oriented to the diagnosis of suppurative thyroiditis with underlying third/fourth branchial arch anomaly. The diagnosis was confirmed with laryngoscopy and the child was treated with endoscopic cauterization. The presence of specific clinical and imaging findings is not definitive, so the diagnosis relies on having a strong suspicion and knowledge of typical locations. So, our purpose is to present the spectrum of relative imaging findings from at least three imaging modalities in order to increase the level of clinical and radiological suspicion of that rare condition and help with accurate diagnosis and planning of the treatment.

## Introduction

Congenital malformations of the branchial arches are the second most common congenital lesions of the head and neck, with second branchial arch anomalies being the most frequently consisting of about 95% of cases and associated in most cases with cysts. On the opposite side, third and fourth arch anomalies are extremely rare representing < 1-5% of cases, and typically manifest as sinus track communicating with the pyriform sinus of the hypopharynx [1,2]. The most common presentation is an inflammatory neck mass commonly on the left involving the thyroid gland. The diagnosis is challenging because there are no pathognomonic clinical and imaging findings and definite surgical treatment.

In our case imaging findings indicative of the diagnosis were detected early even from the initial ultrasound examination and were subsequently confirmed with computed tomography (CT) and magnetic resonance imaging (MRI). We report a case of acute suppurative thyroiditis in a child with congenital fourth branchial arch anomaly and branchial cleft sinus track formation, which consists of a rare condition with only a few relative radiological references published.

## Patient and observation

**Patient information:** a 4-year-old Caucasian boy, was referred to the pediatric emergency room of our hospital complaining of painful neck swelling more prominent on the left side of the neck. From patients´ medical, family, and psychosocial history no significant pathology was referred. No relevant past symptoms were referred.

**Clinical findings:** from clinical examination the region of anterior neck swelling was red, warm, and firm. The general condition of the patient was good. The oropharynx inspection showed no pathologic findings. Respiratory and heart auscultation showed normal findings.

**Timeline of the current episode:** the patient´s parents mentioned intermittent 4 days long fever (until 37.9°C) and sore throat the past few days. Eventually, a painful neck swelling, more prominent on the left side of the neck, occurred from 24 hours.

**Figure 1:**
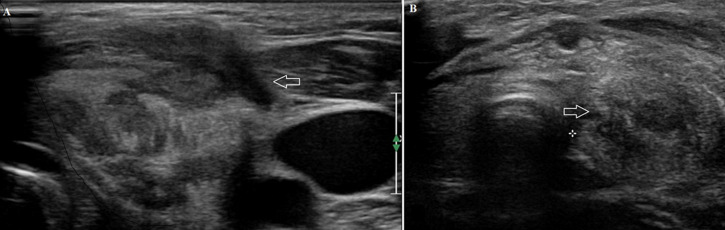
(A, B) first ultrasound imaging assessment, normal thyroid left lobe parenchyma echo texture is replaced from hypoechoic inflammatory lesion, through the findings is noticed a more hypoechoic elongated lesion which corresponds to the sinus track containing air bubbles that cause acousting shadowing

**Diagnostic assessment:** blood tests revealed leukocytarosis, elevated C-reactive protein, and erythrocyte sedimentation rate. A blood culture was sent and was sterile. An electrocardiogram (ECG) examination and thoracic X-ray showed normal findings. The COVID test was negative. The Mantoux test was negative. A serological test for Bartonella was sent with results: IgM 1/80, IgG 1/256. The first sonographic review was performed on the first day of submission and revealed a heterogeneously hypoechoic area with poorly defined margins on the left side of the neck obliterating fat planes and infiltrating the left lobe of the thyroid gland. Moreover, a potential sinus track with serpentine configuration was obvious through the inflammatory region with a measurable lumen diameter (0.5 cm) and the presence of air bubbles in the lumen (Figure 1). On the third day, the patient was submitted for contrast-enhanced CT for better evaluation of the extent of the findings and relation with vital organs such as vessels and trachea. This showed mild right tracheal deviation. The main neck vessels were patent.

**Figure 2:**
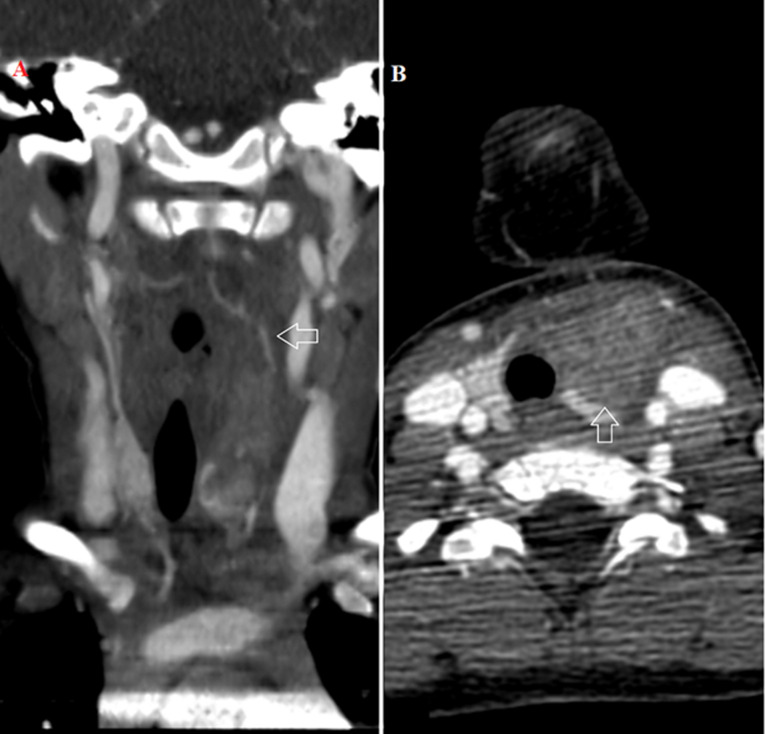
computed tomography with intravenous contrast medium: A) axial imaging heterogeneity in the left thyroid lobe that corresponds to an abscess, the left carotid artery, and internal jugular vein are patent; B) coronal imaging, the inflammatory lesion extends inferior to the hyoid bone to the thoracic inlet, anterior mediastinum fat planes are intact, there is a mild right tracheal deviation

**Figure 3:**
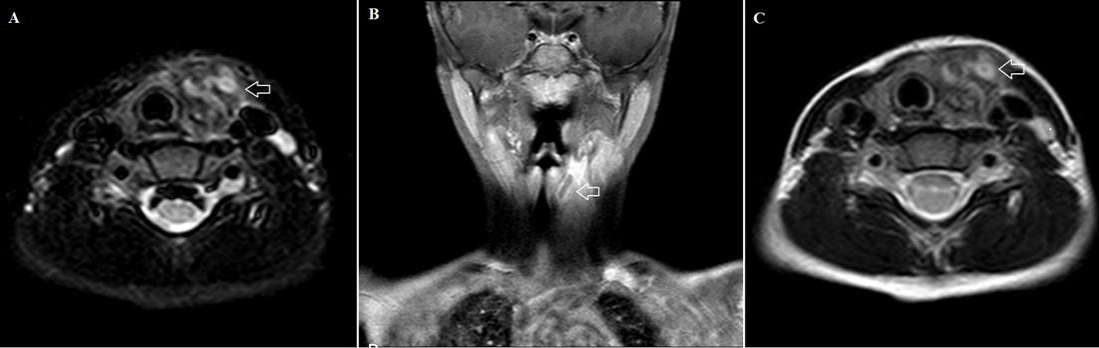
short Tau inversion recovery (STIR), T1-w magnetic resonance imaging (MRI) with contrast administration and T2-w MRI: A) axial STIR image, it is noticed a thin sinus track composed of high signal wall and low signal lumen centrally (air containing); B) coronal T1-w fat sat with paramagnetic iv contrast, the sinus track has distinct enhancing wall, ending near the pharynx; C) axial T2-w image, notice the high signal wall and low signal lumen

**Figure 4:**
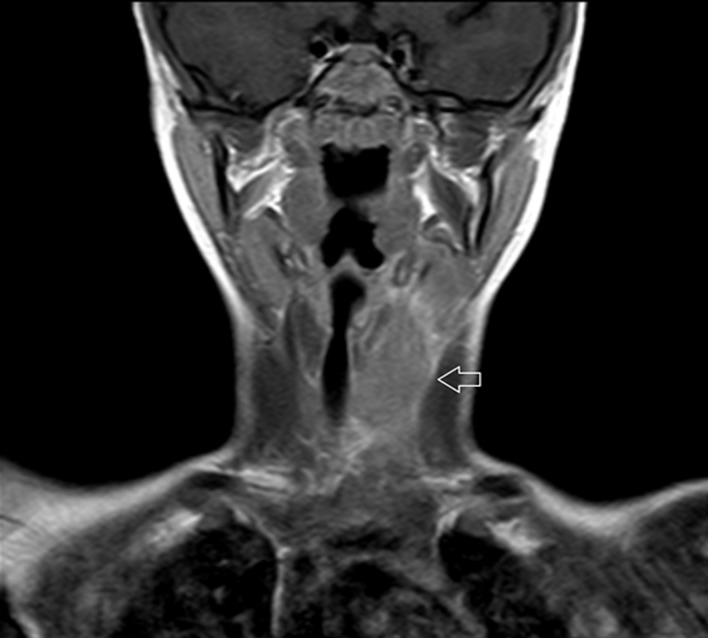
coronal T1-w MRI, enlargement of the left thyroid lobe which has lost normal signal intensity, note obliteration of interposed fat planes with sternocleidomastoid muscle, mild right trachea deviation is obvious, normal flow void is noticed from patent left main neck vessels

**Figure 5:**
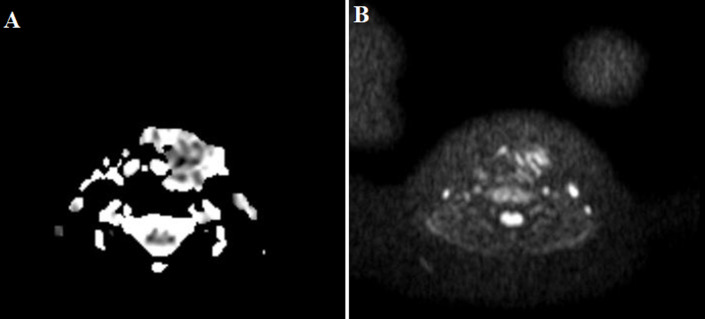
(A, B) apparent diffusion coefficient (ADC) map MRI and diffusion-weighted imaging (DWI); respectively to the configuration of the sinus track on the left is noticed bright signal on DWI and low signal on ADC map, confirming diffusion restriction because of pus content

**Figure 6:**
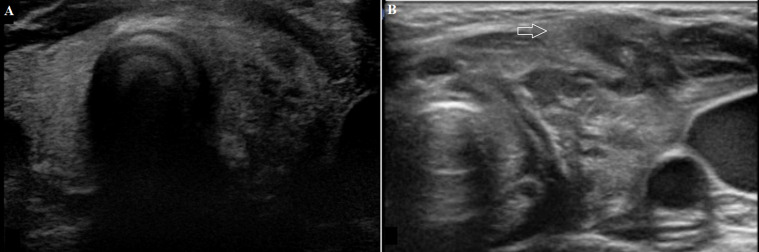
second sonographic evaluation: A) the inflammatory lesion extends between the airway and left carotid artery, centrally the sinus track is obvious; B) axial view of thyroid gland, abnormal enlargement and heterogeneity of left thyroid lobe, compared with normal right thyroid lobe

Anterior mediastinum fat planes were intact (Figure 2). Magnetic Resonance Imaging also revealed better the existence of the sinus track coursing through the infected area from the level of the pharynx to the spatial perithyroideum (Figure 3, Figure 4, Figure 5). Repetitive sonographic evaluation showed even more improvement in the findings in the next few days. No percutaneous evacuation of the abscess was finally necessary (Figure 6).

**Diagnosis:** the differential diagnosis of sonographic findings includes suppurative thyroiditis, subacute thyroiditis, primary thyroid lymphoma, and undifferentiated carcinoma which occasionally present as poorly defined hypoechoic areas [3]. Due to the typical location of clinical and sonographic findings, acute suppurative thyroiditis with underlying third or fourth branchial cleft anomaly was considered the most possible diagnosis.

**Therapeutic interventions:** in our patient the pharyngeal opening of the sinus was endoscopically detected and catheterized. Follow-up and outcome of interventions: in the post-intervention period the patient had no significant pain and was discharged 1 day later in very good general condition. The follow-up pharyngo-laryngospcopy 6 months later showed normal findings. The child three years later had no relapsing episodes.

**Patient perspective:** the patient didn´t mention any significant pain near after the procedure. He returned to his daily activities 9 days after the discharge. He informed us that he felt very relieved and had no recurrent symptoms in the 6-month follow-up.

**Informed consent:** after informing the patient´s parents we managed to take written concern from them in order to be able to publish this case report.

## Discussion

Pathology relative to branchial arches remnants is the second most common congenital lesion of the neck [2,4]. The branchial apparatus is the primary embryologic structure that appears in the fourth week of gestation and gives rise to anatomical structures of the neck [5]. It consists of six clefts lined by ectoderm and six corresponding pouches lined by endoderm [2]. Residual inversion of these primary structures can present as sinuses, fistulas, and cysts [2]. Although second branchial arch remnants most commonly present as cysts, third/fourth arch remnants represent sinus tracks that end at the hypopharynx [1,2].

The sinus track courses usually the left side of the neck according to Adams *et al*. [2] from the pyriform sinus to the upper pole of the left thyroid gland predisposing the spread of infection from the pharynx [1] to an area called spatium perithyroideum where purulent exudative infiltrate left thyroid gland [3]. The laryngeal nerve helps differentiate track´s origin from the third or fourth branchial cleft depending on the course of the track above or below the nerve respectively [2,4]. These are present in early childhood [5]. The most common complication is that of an inflammatory lesion (90%) almost always extending into the thyroid gland [1]. Repeated neck infections or abscesses more often located on the left side with accompanying acute thyroiditis, are more frequently referred from the patient's history. As the orifice of the sinus track is located at the pyriform sinus of the pharynx it predisposes to the spread of upper respiratory infection to spatium perithyroideum. This infection usually follows bouts of upper respiratory infection and is complicated with thyrotoxicosis [1,6].

Imaging techniques such as ultrasound (US), CT, and MRI provide important information relative to the nature, site, and extent of the lesion and its relation with adjacent anatomical structures and the integrity of vital structures such as main neck vessels [1]. The ultrasonography is considered the initial rapid method with no radiation to highlight the inflammatory mass, to confirm the involvement of the thyroid gland, and to check for patency of major neck vessels [6]. It is also a useful tool for evaluation of the response to therapy and for guiding fine needle aspiration of the abscess [6]. A contrast-enhanced computed tomography offers detailed images using thin slices and the ability for 3-D reconstruction and is considered as the modality of choice for demonstrating the site and extent of the lesion [1]. It is also superior to US in detecting anatomic variants and vascularity of the lesions. Moreover, visualization of air within the infected area can increase suspicion of the presence of branchial sinus anomaly as the underlying cause [1]. Magnetic Resonance Imaging is a method without radiation and with high soft tissue contrast resolution that delineates the location, the extent, and the relation of the infectious lesion with the neighboring structures providing supplemental information for accurate preoperative planning [1]. Fistulograms were previously used for diagnosis before the widespread use of CT and MRI [7]. Computed tomography fistulography is a new promising method combining the advantages of modern CT scanner's capabilities for the detection of the sinus track [7].

Complete surgical excision of the sinus track with partial thyroidectomy is considered the radical therapy of this anatomic pathology to avoid recurrent infections [3-5]. As the sinus track is in contiguity with vital structures of the neck, an open surgery could be dangerous. Hopefully, endoscopic therapy using cauterization has great outcomes [2]. All children with suspected clinical and imaging findings with 3^rd^ or 4^th^ branchial cleft anomaly should undergo careful direct pharyngo-laryngospcopy in order to identify internal opening arising from the pyriform sinus apex [1].

## Conclusion

Clinical and imaging findings of arch anomalies are not typical therefore familiarity with that condition increases radiologist´s suspicion for appropriate diagnosis. Recurrent localized neck infection especially on the left in a child should raise suspicion of branchial cleft anomaly. Summarizing, all children with sonographically detected hypoechoic mass on the anterior left neck involving thyroid gland should raise suspicion of branchial anomaly and should undergo careful direct pharyngoscopic examination.
